# Seasonal Release Potential of Sediments in Reservoirs and Its Impact on Water Quality Assessment

**DOI:** 10.3390/ijerph16183303

**Published:** 2019-09-08

**Authors:** Suduan Hu, Tianxiang Wang, Shiguo Xu, Lingxiao Ma, Xinguo Sun

**Affiliations:** 1 Institution of Water and Environment Research, Dalian University of Technology, Dalian 116024, China; 2 China Water Resources Pearl River Planning Surveying & Designing Co, Ltd, Guangzhou 510610, China; 3 Jiangsu Smart Factory Engineering Research Center, Huaiyin Institute of Technology, Huaian 223200, China

**Keywords:** sediments, release, adsorption, seasonal variation, environmental factors, water quality assessment

## Abstract

Reservoir sediments act as important receivers and sources for nutrients in the overlaying water. To determine the sediments adsorption and release characteristics and their impacts on water quality, surface sediments samples collected from Biliuhe reservoir in August 2015 were studied through simulation experiments in a laboratory. The results demonstrated that the equilibrium concentrations (EC_0_) of ammonia nitrogen (NH_4_^+^–N) and total phosphorus (TP) were 8.29 mg/L and 0.025 mg/L, respectively, which were both higher than the average concentrations in the overlying water. Therefore, the sediments of Biliuhe reservoir mainly acted as the pollution sources for NH_4_^+^–N and TP, and the release to water showed a seasonal variation. When potential release amounts were considered, the average concentrations of NH_4_^+^–N and TP in the overlaying water could reach 6.4 mg/L and 0.21 mg/L, respectively, which significantly exceeded the current contents. Further, water quality exhibited a decreased tendency after taking the release potential of nutrients into account of water quality assessment. Among the samples, 42% and 33% of them in summer and autumn exceeded the third level of the National Surface Water Quality Standards of China (GB3838-2002). The results indicated that sediments release potential had an unignored influence on water quality during various seasons.

## 1. Introduction

As an important component of the reservoir water environment, sediments have the contradictory characteristics of “source–sink” for pollutants. During the long operation period of a reservoir, sediments have been receivers of various pollutants including point and nonpoint sources [1,2]. With the perennial accumulation of pollutants and mineralization of sedimentary organic matter, concentrations of a variety of constituents in sediments are many times greater than those in water. When the external environment condition is changed, constituents will be released from sediments into the water column through desorption, dissolution, mineralization, and microbe release, leading to algae over-blooming and water quality degradation [3,4,5,6]. Thus, sediments act as pollution sources to water.

Previous studies have indicated that phosphorus and nitrogen released from sediments are the main determinants of eutrophication following reductions of external loadings. For example, in West Lake of Hangzhou, the calculated release capacity of phosphorus from sediments accounted for 36.4% of the average external phosphorus loading [7]. A study in Lake Erken, Sweden, showed 99% of the nutrients were from sediments in summer [8]. It is also pointed out that for some eutrophic lakes and reservoirs, the release of nutrients such as nitrogen and phosphorus from sediments will still cause eutrophication, and this effect may last for decades [9]. An increasing amount of studies have shown that reservoir sediments can also be sources of pollution after decades of operation [10,11,12,13]. Therefore, the release of sediments is gradually considered in water quality assessment [14]. It is proven that pollutants released from sediments can affect the water quality evaluation results and sediments release is a non-negligible factor in water quality simulation and evaluation [15,16].

The adsorption and release characteristics of sediments change with the contents of the overlying water, physicochemical properties of sediments [17,18], water temperature (T_W_) [19], dissolved oxygen (DO) [20], pondus hydrogenii (pH) conditions [21], and other environmental factors [22,23]. For reservoirs in the monsoon climate zone, environmental and water quality indicators vary with seasonal changes, which cause the adsorption and release characteristics of the sediments to change seasonally. For instance, high temperature and high heat in summer can easily lead to the formation of the thermocline in water, which will give rise to the release of pollutants in the sediments. In the summer of 2008, Tanghe reservoir in Liaoning province suffered an unexpected water supply incident as a result of the release of hydrogen sulfide from sediments under the influence of the summer thermocline [24]; Xinlicheng reservoir in Jilin province encountered eutrophication because of the excess nitrogen and phosphorus released from the sediments in the flood season of 2007 and 2008 [25]. Therefore, reasonable and accurate evaluation of the sediments release potential and its impact on water quality is an important basis for the development and utilization of water resources, especially taking into account the specific seasonal variation of external environmental factors.

Biliuhe reservoir was built in 1975 and is now the largest water source of Dalian, making up 80% of the water supply for Dalian. The nutrients in the water column, such as the total nitrogen (TN), total phosphorus (TP), and ammonia nitrogen (NH_4_^+^–N), may exceed third level of the National Surface Water Quality Standards of China (GB3838-2002) at times, resulting in the risk of water eutrophication [26]. The TN and TP contents in sediments have reached 2725 mg/kg and 571mg/kg after nearly 30 years of operation [27,28]. Compared with other similar reservoirs in China, the sediments have a higher release risk for nitrogen and phosphorus. Besides, Biliuhe reservoir belongs to the monsoon climate zone, and the environmental factors such as T_W_, DO, and pH have obvious seasonal variation laws in accordance with the monsoon climate. As a consequence, to evaluate the sediments release potential in combination with different seasonal conditions and the effects on water quality can provide support for the safety of water supply, which is of significance. Therefore, this paper carried out a series of physical simulation experiments to identify the adsorption and release characteristics of the sediments in Biliuhe reservoir, and explored the effects of different environmental factors on the release characteristics. Then, the sediments potential release amounts of NH_4_^+^–N and TP in different seasons were calculated in combination with the environmental factors in actual operating conditions. Finally, the seasonal variation of water quality considering the sediments release potential was assessed.

## 2. Materials and Methods

### 2.1. Study Area and Sampling

**Figure 1:**
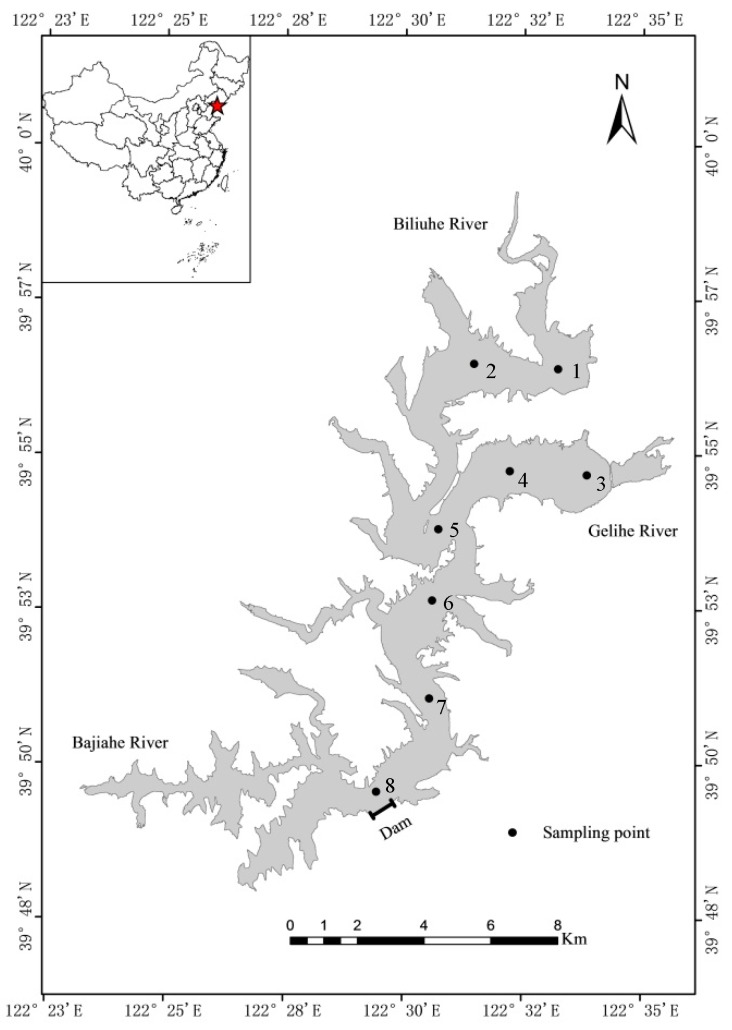
Location of the study area and sampling points.

This paper takes Biliuhe reservoir in Dalian, Northern China as an example. As shown in Figure 1, Biliuhe reservoir has a mainstream, Biliuhe River, and two main tributaries, Gelihe River and Bajiahe River. According to the “Regulation for Water Environmental Monitoring of China (SL 219-2013)”, eight sampling points were set along the longitudinal section of the reservoir from the main inflow entrance (Sampling points 1 and 2), the Gelihe River inflow entrance (Sampling points 3 and 4), to the center of the reservoir (Sampling points 5, 6, and 7), and to the front of the dam (Sampling point 8). The average water depth is 12.8 m, so the stratified water samples were collected at 5 m intervals using Niskin bottles from January 2013 to December 2016 at the beginning of each month. The surface sediments samples were collected at the sampling points by use of a Van Veen Grab Sampler in August 2015. At the same time, T_W_, DO, and pH were detected on-site by WTW Multi 340i. The samples were carried to the laboratory soon after the sampling. There, water samples were detected soon by reference methods in the “Water and Wastewater Monitoring and Analysis Method (Fourth Edition), Beijing: China Environmental Science Press, 2002”. Besides, the sediments were dried in natural wind, smashed and screened through a mesh size of 100, and finally stored in a freezer at a temperature of −20 °C until analysis [29]. Statistical analysis was performed using SPSS 23. 0 (IBM, Armonk, NY, USA).

### 2.2. Experimental Methods

Nitrogen and phosphorus are the main determinants of water eutrophication. Previous experiments and studies have shown that NH_4_^+^–N is the main exchange fraction of nitrogen at the sediment–water interface [30,31]. Thus, NH_4_^+^–N and TP were chosen as the two indices for the sediments experimental simulation. Besides, in the laboratory, all sampled sediments were pooled together and thoroughly homogenized.

#### 2.2.1. Sediments Adsorption Experiments

NH_4_^+^–N solutions with gradient concentrations of 0, 1, 5, 10, 15, 25, 30, 40, 50, 60, 70, 90, and 100 mg/L were prepared by ammonium chloride, and the pH of all of them was adjusted to 7.0 by 0.01 mol/L HCL and 0.01 mol/L NaOH [4]. In a series of 100 mL centrifuge tubes, 1 g of sediments was placed and treated with 100 mL NH_4_^+^–N solution at various concentrations. Thereafter, the tubes were shaken for 24 h at the temperature of 25 °C in a stable temperature water shaking bath at 200 rpm. After 24 h of equilibration [32], the samples were centrifuged at 5000 rpm for 10 min and then filtered through a 0.45 um filter membrane. The filtrate was taken for NH_4_^+^–N analyses via Nessler’s reagent spectrophotometry [33]. Triplicate experiments were carried out at the same time, and the relative error was lower than 5%.

A series of initial TP concentrations of 0, 0.5, 1, 2, 5, 8, 10, 15, 25, 30, and 50 mg/L were prepared by potassium dihydrogen phosphate. The pH of the solutions was also maintained at 7.0 by adjusting with 0.01 mol/L HCL and 0.01 mol/L NaOH [34]. Similarly, 1 g sediments samples and 100 mL TP solution of different concentrations were put into 100 mL centrifuge tubes. The other experimental conditions were the same as those of the NH_4_^+^–N adsorption experiments. TP remaining in the filter was analyzed by the phosphor molybdenum blue spectrophotometry [33]. Triplicate experiments were carried out at the same time, and the relative error was lower than 5%.

In the end, the adsorption capacity was calculated to describe the sediments adsorption effect of NH_4_^+^–N (TP). The equation is as follows [35].

(1) $$q=\frac{\left(C_{0}-C_{e}\right)\times V}{W},$$

where *q* is adsorption capacity of NH_4_^+^–N (TP), mg/kg; *C_0_* is the initial concentration of NH_4_^+^–N (TP) in the solution, mg/L; *C_e_* is the equilibrium concentration of NH_4_^+^–N (TP) in the solution, mg/L; *V* is the volume of the solution, mL; and *W* is the mass of the sediments, g.

#### 2.2.2. Sediments Release Experiments

NH_4_^+^–N stock solution was prepared by ammonium chloride, and TP stock solution was prepared by potassium dihydrogen phosphate. Firstly, a series of 1 g sediments samples was added into 100 mL centrifuge tubes. Then, a sequence of low concentrations of NH_4_^+^–N solutions (0, 0.5, 1, 2, 4, 6, 8, 10 mg/L) prepared by NH_4_^+^–N stock solution was separately put into the above centrifuge tubes [36]. Meanwhile, a sequence of low concentrations of TP solutions (0, 0.0025, 0.005, 0.0125, 0.020, 0.025, 0.030, 0.040 mg/L) prepared by TP stock solution was separately put into 100 mL centrifuge tubes with 1 g sediments samples [35]. Next, the experimental conditions were kept the same as in the former experiments and the release amounts of NH_4_^+^–N (TP) at different initial concentrations were calculated according to Equation (1). Triplicate experiments were carried out at the same time, and the relative error was lower than 5%.

#### 2.2.3. Sediments Release Experiments under Different T_W_, DO, and pH

To study sediments release characteristics under different T_W_, 1 g sediments samples and 100 mL distilled water were put into 100 mL centrifuge tubes. Afterwards, the tubes were separately shaken at the temperatures of 5, 15, and 25 °C in stable temperature water shaking baths at 200 rpm. After 24 h of equilibration, the samples were centrifuged and filtered, and then the supernatants were taken for analysis.

Similarly, an anaerobic environment (DO = 2 ± 0.5 mg/L), low oxygen environment (DO = 4 ± 0.5 mg/L), and natural environment condition (DO = 8.5 ± 0.5 mg/L) were simulated by the boiling method to study the release characteristics under different DO conditions. In addition, different pH gradients (2, 7, 11) were simulated by use of 0.01 mol/L NaOH and 0.01 mol/L HCl solutions to conduct the release experiments under different pH conditions. Triplicate experiments were carried out at the same time, and the relative error was lower than 5%.

### 2.3. Water Quality Assessment Methods Considering Sediments Release Potential

The variable fuzzy pattern recognition (VFPR) model was selected to assess the water quality because of its superiority in assessing the index with continuity and uncertainty characteristics [37].

#### 2.3.1. Assessment Index System

Firstly, the water index system *X*’*_ij_* (*i* is the number of indicators and *j* is the number of samples) was set up according to the principles of comprehensiveness, systematicness, and availability. In this paper, eight indicators including DO, pH, TP, total nitrogen (TN), NH_4_^+^–N, iron (Fe), manganese (Mn), and permanganate index (COD_Mn_) were selected for water quality assessment. These indicators explained the water quality from the aspects of comprehensive, eutrophication, and heavy metals, so the established index system could effectively represent the water quality of the reservoir.

At the same time, the release potential $\Delta x_{i}$ of NH_4_^+^–N and TP in sediments under different environmental conditions was calculated by Equation (2) according to the sediments release experiments:

(2) $$\Delta x_{i}=\frac{Q\times q_{i}}{h_{i}\times 100},$$

where Δ*x_i_* is the increment of the index *i*, mg/L; *Q* is the sediments content per unit area, g/cm^2^; *q_i_* is the release amount of index *i*, mg/kg; and *h_i_* is the water depth of index *i*, m.

Thus, the new assessment index system *X_ij_* is obtained:

(3) $$X_{ij}={X^{\prime }}_{ij}+\Delta X_{ij}.$$

#### 2.3.2. Indicator Normalization

In order to eliminate the dimension of the index and unify the change direction of the indicator (TN, TP and other inverse index, DO, transparency, and other positive index), water quality samples *X_ij_* and water quality standards *Y_ic_* were normalized using Equation (4) and (5), respectively.

(4) $$r_{ij}=\{\begin{array}{ccc} 0 & , & x_{ij}\geq y_{ic}(\mathrm{inverse}\;\mathrm{index})\mathrm{or}x_{ij}\leq y_{ic}(\mathrm{positive}\;\mathrm{index})\\ \frac{x_{ij}-y_{ic}}{y_{i1}-y_{ic}} & , & y_{i1}<x_{ij}<y_{ic}(\mathrm{inverse}\;\mathrm{index})\mathrm{or}\;y_{i1}>x_{ij}>y_{ic}(\mathrm{positive}\;\mathrm{index})\\ 1 & , & x_{ij}\leq y_{i1}(\mathrm{inverse}\;\mathrm{index})\mathrm{or}x_{ij}\geq y_{i1}(\mathrm{positive}\;\mathrm{index}) \end{array}\;,$$

(5) $$s_{ih}=\{\begin{array}{ccc} 0 & , & y_{ih}=y_{ic}(\mathrm{positive}\;\mathrm{index}\;\mathrm{or}\;\mathrm{inverse}\;\mathrm{index})\\ \frac{y_{ih}-y_{ic}}{y_{i1}-y_{ic}} & , & y_{i1}<y_{ih}<y_{ic}(\mathrm{positive}\;\mathrm{index})\mathrm{or}\;y_{i1}>y_{ih}>y_{ic}(\mathrm{inverse}\;\mathrm{index})\\ 1 & , & y_{ih}=y_{i1}(\mathrm{positive}\;\mathrm{index}\;\mathrm{or}\;\mathrm{inverse}\;\mathrm{index}) \end{array},$$

where *c* is the highest level of the standard, *c* = 1,2,3,4,5; *r_ij_* and *s_ih_* are the normalized results of the samples *X_ij_* and water quality standards *Y_ic_*; and *h* is the standard level of water quality (*h* = 1,2,... c).

#### 2.3.3. Comprehensive Relative Membership Degree

Equation (6) is used to calculate the comprehensive relative membership degree *u_hj_* of water quality sample *j* belonging to the water quality standard *h*:

(6) $$u_{hj}=\{\begin{array}{cc} 0 & h<a_{j}\mathrm{or}h>b_{j}\\ \frac{1}{\sum_{k=a_{j}}^{b_{j}}{\left\{{\frac{\sum_{i=1}^{m}\left[w_{ij}\left(r_{ij}-s_{ih}\right)\right]}{\sum_{i=1}^{m}{\left[w_{ij}\left(r_{ij}-s_{ik}\right)\right]}^{p}}}^{p}\right\}}^{\frac{a}{p}}} & a_{j}\leq h\leq b_{j}\\ 1 & h=a_{j}=b_{j} \end{array},$$

where *a_j_* and *b_j_* are the minimum and maximum value of water quality sample *j*, respectively; *w_ij_* is the weight of index *i* of water quality sample *j*, $\sum_{i=1}^{m}w_{i}=1$; *a* is the optimization criterion parameter, *a* = 1 represents the linear criterion and *a* = 2 represents the least-squares criterion; and *p* is the distance parameter, *p* = 1 represents Hamming distance and *p* = 2 represents Euclidean distance.

#### 2.3.4. Comprehensive Assessment Level of Water Quality

When the comprehensive relative membership degree *u_hj_* is calculated, the comprehensive assessment level of water quality sample *j* can be obtained using Equation (7).

(7) $$H=\sum_{h=1}^{c}u_{\mathrm{hj}}h,$$

where *H* is the characteristic value of water quality and *h* is the standard level of water quality (*h* = 1,2,... c).

## 3. Results and Discussion

### 3.1. Identification of Source–Sink Characteristics of Sediments

#### 3.1.1. Sediments NH_4_^+^–N Source–Sink Identification

**Figure 2:**
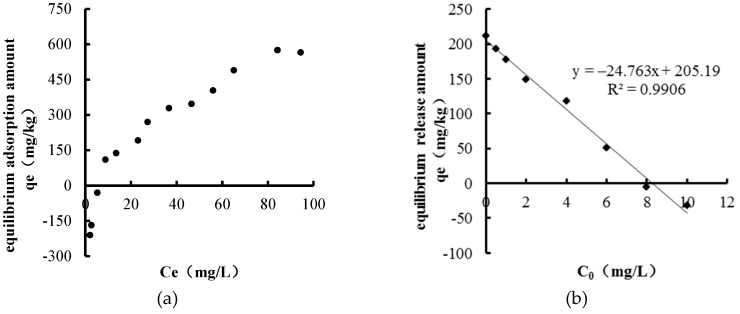
Characteristics of NH_4_^+^–N adsorption and release in sediments of Biliuhe reservoir: (**a**) NH_4_^+^–N adsorption characteristic; (**b**) NH_4_^+^–N release characteristic.

The characteristics of NH_4_^+^–N adsorption and release in sediments of Biliuhe reservoir are shown in Figure 2.

It can be seen from the adsorption characteristic (Figure 2a) that sediments began to adsorb NH_4_^+^–N from the overlying water when its concentration was between 5~10 mg/L, and the adsorption amount increased with the increase of the NH_4_^+^–N concentration in the overlying water. When the concentration of NH_4_^+^–N in the overlying water was 80~100 mg/L, the adsorption reached equilibrium.

The Langmuir and Freundlich equations were introduced to describe the NH_4_^+^–N adsorption characteristic, which have both been widely used to describe the adsorption behavior [38,39].

**①**The Langmuir equation assumes that the adsorbed particles are completely independent, and the equation is as follows [40]:

(8) $$q_{e}=q_{L}\frac{K_{L}C_{e}}{1+K_{L}C_{e}}.$$

Also, this equation could be expressed by a linear model as follows:

(9) $$\frac{1}{q_{e}}=\frac{1}{q_{L}•K_{L}}\frac{1}{C_{e}}+\frac{1}{q_{L}},$$

where *C_e_* is the equilibrium concentration of the overlying water, mg/L; *q_e_* is the equilibrium adsorption amount, mg/kg; *q_L_* is the theoretical saturation adsorption capacity, mg/g; and *K_L_* is the Langmuir constant related to the adsorption heat, L/mg.

**②**The Freundlich equation is more widely used to describe the multilayer sorption on a heterogeneous surface, and the equation is as follows [41]:

(10) $$q_{e}=kC_{e}^{1/n}.$$

Also, this equation could be expressed by a linear model as follows:

(11) $$\mathrm{lg}q_{e}=\frac{1}{n}\mathrm{lg}C_{e}+\mathrm{lg}k,$$

where *q_e_* is the equilibrium adsorption amount, mg/kg; *C_e_* is the equilibrium concentration of the overlying water, mg/L; and *k* and *n* are both the Freundlich constants related to the capacity of adsorption substrate.

The calculation results (Table 1) showed that the Langmuir and Freundlich equations well fitted the NH_4_^+^–N adsorption characteristic with R^2^ of 0.9735 and 0.9846, respectively. The theoretical saturation adsorption capacity of NH_4_^+^–N was 909.1 mg/kg.

As can be seen in Figure 2b, with the increase of initial NH_4_^+^–N concentration in the overlying water, the release amount decreased inversely, and sediments began to adsorb NH_4_^+^–N from the overlying water when the initial NH_4_^+^–N concentration was between 8 and 10 mg/L. The amounts of NH_4_^+^–N released from sediments had a linear relationship with the initial concentration (R^2^ = 0.9906) [42]. Therefore, the equilibrium concentration (EC_0_) [43] of NH_4_^+^–N was obtained as 8.29 mg/L. The results are shown in Table 1.

The annual average concentration of NH_4_^+^–N in Biliuhe reservoir was 0.28 mg/L from 2013 to 2016, and the seasonal average values during winter, spring, summer, and autumn were 0.27, 0.23, 0.32, and 0.31 mg/L, respectively. The NH_4_^+^–N concentration of Biliuhe reservoir was much lower than the EC_0_ of 8.29 mg/L, indicating that the sediments of Biliuhe reservoir had a high risk of release. Therefore, the sediments of Biliuhe reservoir will function as a pollution source for NH_4_^+^–N.

#### 3.1.2. Sediments TP Source–Sink Identification

**Figure 3:**
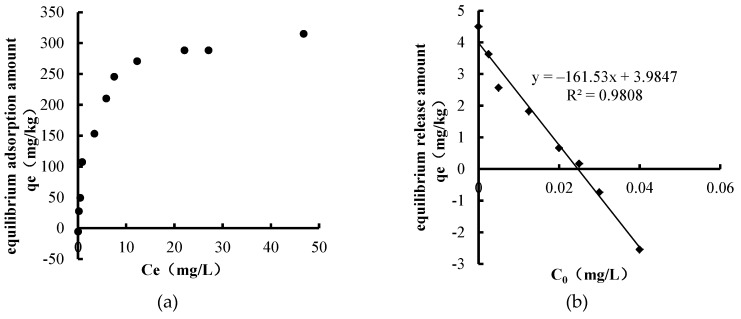
Characteristics of total phosphorus (TP) adsorption and release in sediments of Biliuhe reservoir: (**a**) TP adsorption characteristic; (**b**) TP release characteristic.

The characteristics of TP adsorption and release in sediments of Biliuhe reservoir are shown in Figure 3.

It can be seen from the adsorption characteristic (Figure 3a) that sediments began to adsorb TP from the overlying water when its concentration was between 0~0.05 mg/L, after which the adsorption amount increased with the increase of TP concentration in the overlying water. When the concentration of TP in the overlying water was 20 mg/L, the adsorption reached equilibrium. Similarly, both the Freundlich equation and the Langmuir equation provided a good fit to the TP adsorption characteristic with R^2^ of 0.9899 and 0.9117, respectively. The theoretical saturation adsorption capacity of TP was 312.5 mg/kg (Table 2).

The TP release characteristic (Figure 3b) showed that the amount of TP released from sediments and the initial concentration of TP in solution exhibited a linear relationship (R^2^ = 0.9808). The calculated EC_0_ of TP was 0.025 mg/L. The fitting results are shown in Table 2.

EC_0_ was higher than the annual average concentration of TP (0.024 mg/L) in overlying water from 2013 to 2016, indicating the risk that TP releases to overlying water is high. The seasonal average values of TP concentration in the overlaying water during winter, spring, summer, and autumn were 0.019, 0.020, 0.035, and 0.021 mg/L, respectively. Among these, the TP concentration in summer exceeded the EC_0_, which indicated that the reservoir sediments could adsorb TP from the overlying water in summer. In conclusion, the sediments of Biliuhe reservoir had the dual characteristic of source and sink for TP.

### 3.2. Sediments Release Characteristics under Different T_W_, DO, and pH

#### 3.2.1. Water Temperature

**Figure 4:**
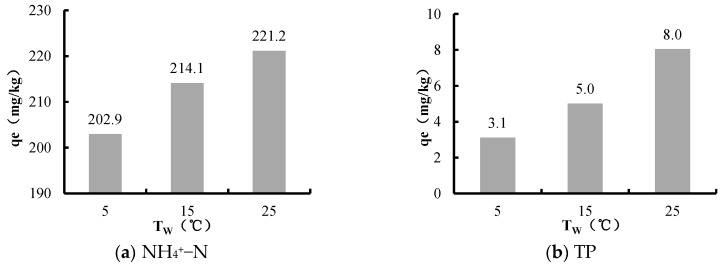
The relation between T_W_ and the release amounts of NH_4_^+^–N and TP in Biliuhe reservoir.

Figure 4 shows the relation between water temperature (T_W_) and the release amounts of NH_4_^+^–N and TP.

The experiment results showed that the release amounts of NH_4_^+^–N and TP increased with T_W_. At 25 °C, the release amounts of NH_4_^+^–N and TP from sediments were 1.1 and 2.6 times greater than that at 5 °C.

T_W_ is an important factor affecting soil mineralization. When T_W_ increased, more organic nitrogen was transformed to ammonium nitrogen as the soil mineralization strengthened, which promoted NH_4_^+^–N to release from sediments. Meanwhile, the reduced oxygen caused by the strengthening soil mineralization impeded the nitrification reaction in the sediments. Under the integrated influences, the NH_4_^+^–N released from sediments increased with the temperature. The results from previous experiments in Chaohu Lake also suggested the same trend [44].

An increase of T_W_ would promote the migration of phosphorus in sediments through pore water to overlaying water as the sediments adsorption capacity of TP was decreased [45,46]. At the same time, the activity of the microorganisms was also enhanced, and this effectively promoted the conversion of organic phosphorus to inorganic phosphate [47,48]. In addition, the consumption of oxygen by the microorganisms caused the dissolved oxygen to decrease. Further, the decreased oxygen caused the redox potential decline, and Fe^2+^ transformed from Fe^3+^ increase; therefore, a large amount of Fe–P was released from sediments.

#### 3.2.2. Dissolved Oxygen

**Figure 5:**
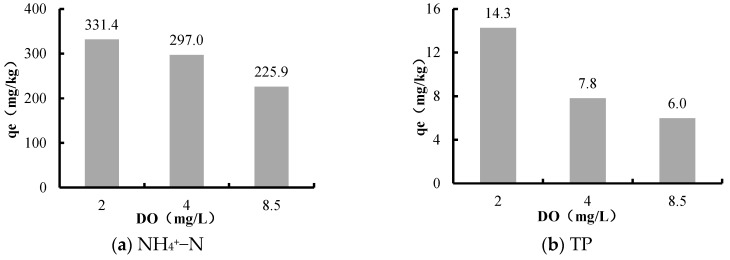
The relation between dissolved oxygen (DO) and the release amounts of NH_4_^+^–N and TP in Biliuhe reservoir.

The release amounts of NH_4_^+^–N and TP under anaerobic environment (DO = 2 ± 0.5mg/L), low oxygen environment (DO = 4 ± 0.5mg/L), and natural environment conditions (DO = 8.5 ± 0.5mg/L) are shown in Figure 5.

The results showed that there was an inverse relation between the release amount and the DO. The release amounts of NH_4_^+^–N and TP during the anaerobic environment was 1.5 and 2.4 times greater than that in the natural state, indicating that low DO could dramatically promote the release of NH_4_^+^–N and TP in sediments.

Under low DO circumstances, the nitrification reaction in sediments was weakened, while the ammonization reaction was enhanced instead. This promoted the release of NH_4_^+^–N in sediments. Therefore, the release amount of NH_4_^+^–N in the low DO environment was higher than that in the aerobic environment.

In addition, the experiment system was in the oxidation environment because of sufficient DO. In the oxidation environment, metal ions, such as iron and manganese, were oxidized to their higher states (FeIII and MnIV). These metal minerals were combined with phosphorus to form compounds that were difficult to dissolve into water [20]. Thus, the release amount of TP from sediments was decreased. Conversely, the iron and manganese precipitates will dissolve and release PO_4_^3−^ under the hypoxic environment [49]. Therefore, the release intensity and speed of TP were higher under hypoxic environment than that in the aerobic environment.

#### 3.2.3. pH

**Figure 6:**
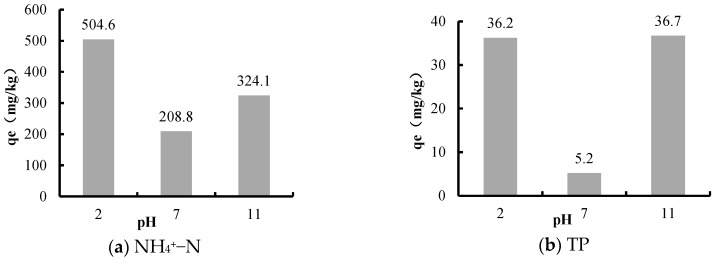
The relation between pH and the release amounts of NH_4_^+^–N and TP in Biliuhe reservoir.

Through the sediments release experiments under different pH, the release amounts of NH_4_^+^–N and TP were obtained, and are shown in Figure 6.

pH plays a vital role in the release process of NH_4_^+^–N and TP. The experiment results showed that the release amounts of NH_4_^+^–N and TP were both in a “U” type relation with the pH of the overlying water. Specifically, both acidic and alkaline conditions could promote the release of NH_4_^+^–N and TP from sediments, while the release amount was the minimum under neutral condition.

Under acidic conditions, the increased H^+^ in the water interacted with the NH_4_^+^ adsorbed by the colloidal deposits, and more NH_4_^+^ were gradually released from sediments into the overlying water. In the alkaline environment, the increased OH^-^ reacted chemically with NH_4_^+^ and produced NH_3_, thus more NH_4_^+^–N was rapidly released from sediments in the form of NH_3_. A previous study showed that 99% of all ammonium nitrogen was in the form of NH_4_^+^ when the pH of water body was 7.3, and more than 90% of the ammonium nitrogen existed in the form of NH_3_ when the water pH was 10.3 [50], so the release amount of NH_4_^+^–N was the minimum under neutral conditions. This was consistent with the NH_4_^+^–N release characteristics of the Wabu Lake sediments [51].

The effect of pH on the release of phosphorus from sediments was mainly the result of the fact that pH could affect the adsorption and ion exchange between phosphorus and sediments [52]. Under alkaline conditions, the P-binding capacity of Fe–P and Al–P was decreased, OH^−^ reacted with inorganic phosphorous such as Fe–P and Al–P to form HPO_4_^2-^, which promoted the release of phosphorus from sediments [53,54]. Under acidic conditions, the clay minerals’ surface negative charge was reduced, and various compounds including Ca–P became more soluble [55]. This promoted the release of phosphorus from sediments, while phosphorus ions mainly existed in the form of phosphates under neutral conditions, and these phosphates were easily combined with metal cations to form precipitates. As a result, the TP release amount from sediments was the lowest under neutral conditions. This was similar to the TP release characteristics of sediments in Wabu Lake [51].

### 3.3. Seasonal Variations of Sediments Release Potential

#### 3.3.1. Seasonal Variations of Environmental Factors in Biliuhe Reservoir

**Figure 7:**
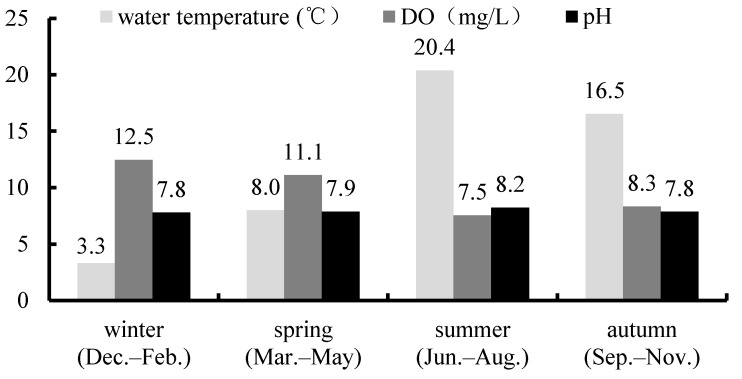
The seasonal variations of influenced factors in Biliuhe reservoir.

The seasonal variation characteristics of T_W_, DO, and pH in Biliuhe reservoir are shown in Figure 7.

It can be seen from Figure 7 that T_W_, DO, and pH of the reservoir all had obvious seasonal variation characteristics.

T_W_ is one of the important factors affecting the physical, chemical, and biological reactions in water and sediments. Biliuhe reservoir is located in the seasonal climate region of Northern China, so its T_W_ has obvious seasonal variation with the seasonal change of air temperature. In winter (December–February), the T_W_ of the reservoir is the lowest with the monthly average value of 3.3 °C. During this period, there will be a frozen period of two to three months per year. Afterwards, T_W_ begins to rise in spring (March–May), and reaches the maximum of 20.4 ℃ throughout the year in summer (June–August). While in autumn (September–November), T_W_ starts to decrease again.

Similar to T_W_, DO in Biliuhe reservoir also shows an obvious seasonal variation with the highest in winter and the lowest in summer. In winter, DO reaches the annual maximum value of 12.6 mg/L because of the low T_W_ and the barrier of ice. As T_W_ increases from spring, the consumption of DO also increases because of the accelerated physical and chemical reactions in water and sediments. So DO content of the reservoir gradually decreases after winter. Generally, DO drops to the lowest in summer and the value is about 7.5 mg/L, but DO could even fall to about 2 mg/L in the bottom layer owing to the existence of the thermocline. After summer, with the decrease of T_W_, the biological activity in water decreases. While the oxygen enrichment ability starts to increase, so the DO begins to increase slowly in the autumn.

pH is an important factor affecting various chemical reactions in water and sediments. The pH of the reservoir changes little throughout the year with the value between 7 and 9, but the pH is higher in summer. In summer, high T_W_ promotes algae growth and photosynthesis. With more CO_2_ in the water being absorbed, pH consequently increases slightly. As it comes to winter, the low T_W_, the barrier of ice, and the coverage of snow together weaken the photosynthesis in the water, so the pH of the water in winter is lower than that in summer. Meanwhile, in spring and autumn, the photosynthesis in water is not significantly increased, so the pH of water is close to winter.

The environmental indicators change in circles and naturally form the specific seasonal variation characteristics of reservoirs in Northern China, that is, low T_W_, high DO, and low pH in winter; high T_W_, low DO, and high pH in summer; and transitional variation in spring and autumn [56,57].

#### 3.3.2. Sediments Release Potential in Different Seasons

According to the relation between the simulated release amount of sediments and environmental factors in Section 3.2, the release amounts of NH_4_^+^–N and TP in the sediments of Biliuhe reservoir under actual environmental conditions were calculated by the interpolation method. Specifically, for one sample, the maximum value of the respective release amounts under its T_W_, DO, and pH was taken for the sediments release potential.

The field investigation indicated that the average sedimentation depth of the Biliuhe reservoir was 40 cm, the average dry density of the sediments was 1.55 g/cm^3^, and the average moisture content was about 180%. Then, the sediments content per unit area was calculated as 34.4 g/cm^2^. According to the monitored water depth of the Biliuhe reservoir, the impact of sediments release potential on water, that is, the sediments increment, could be calculated by Equation (2).

**Figure 8:**
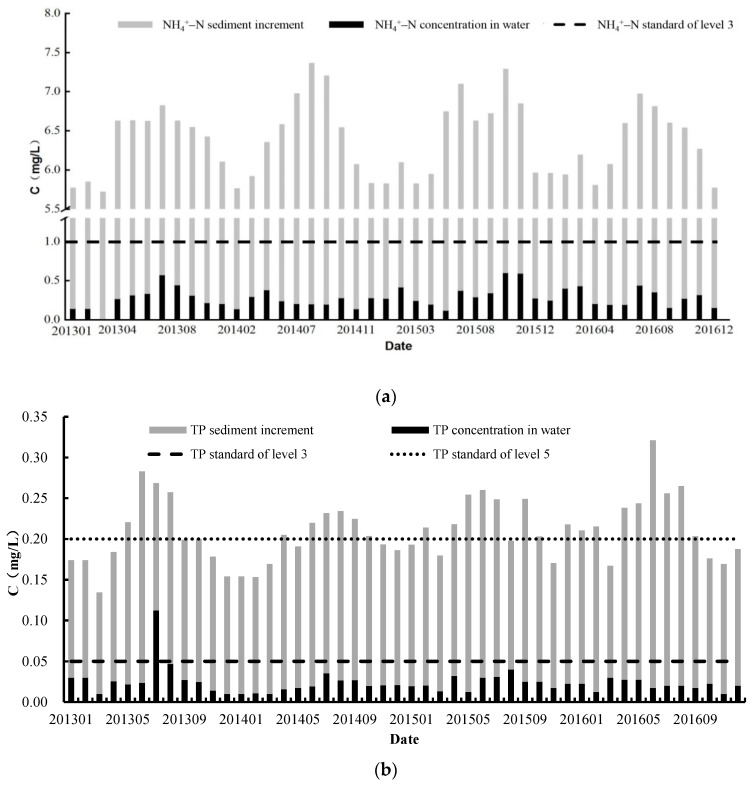
The sediments increment of NH_4_^+^–N and TP of Biliuhe reservoir from 2013 to 2016: (**a**) the sediments increment of NH_4_^+^–N; (**b**) the sediments increment of TP.

The calculated sediments increment of NH_4_^+^–N and TP of Biliuhe reservoir from 2013 to 2016 was shown in Figure 8.

It can be seen from Figure 8 that the annual average NH_4_^+^–N and TP in the water of Biliuhe reservoir from 2013 to 2016 were 0.28 mg/L and 0.024 mg/L, respectively, of which the levels were both less than the third level of water quality standards (GB3838-2002).Unexpectedly, however, the TP was 0.113 mg/L in July 2013, which exceeded the fourth level (0.1 mg/L). When the release potential of sediments was considered, the average amount of NH_4_^+^–N in the reservoir reached 6.4 mg/L, which was 22 times the average concentration in the water and far exceeded the fifth level (2.0 mg/L) of the water quality standards. Meanwhile, the average value of TP changed from 0.024 mg/L to 0.21 mg/L, and increased by eight times the average in the water. By comparing with the water quality standards, the average TP of the reservoir also exceeded the fifth level (0.20 mg/L). This indicated that reservoir sediments had a strong potential to release NH_4_^+^–N and TP, which posed a great threat to the reservoir water quality.

At the same time, the concentrations of NH_4_^+^–N and TP were the highest in summer and the lowest in winter. Similarly, the sediments increment of NH_4_^+^–N and TP also showed seasonal variations, with the highest concentration during summer and the lowest during winter. This could be attributed to the seasonal variations of T_W_, DO, and pH of the reservoir. As described in Section 3.2, high T_W_, low DO, and an acid or alkaline condition all promoted the release of NH_4_^+^–N and TP from sediments. From winter to summer, T_W_ and pH kept increasing, while DO kept decreasing all along. These factors together contributed to the highest release amount of NH_4_^+^–N and TP in summer. When it came to the end of summer, T_W_ started to decline, while DO increased, which weakened the release capacity of NH_4_^+^–N and TP from sediments. Furthermore, the average pH of the reservoir in autumn, winter, and spring was 7.8, which was much closer to the neutral condition, so the effect of pH on the release of sediments was not obvious. Therefore, the sediments increment of NH_4_^+^–N and TP began to decrease gradually from autumn, and reached the lowest in winter.

### 3.4. Seasonal Assessment of Water Quality Considering Sediments Release Potential

**Figure 9:**
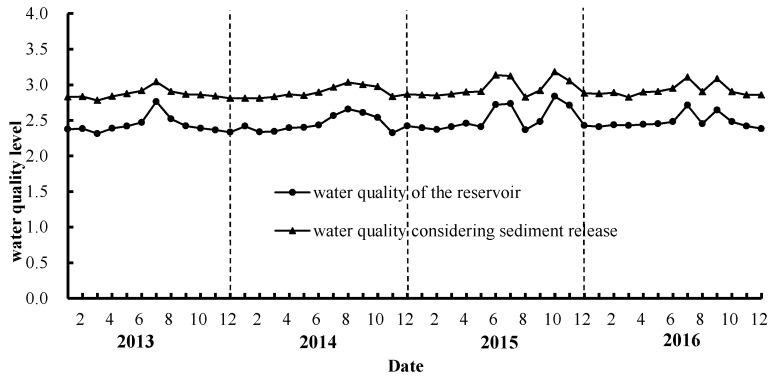
Water quality assessment results considering the release potential of sediments from 2013 to 2016 in Biliuhe reservoir.

The assessment index system considering sediments release potential *X_ij_* was obtained using Equation (3), and the weight of each indicator was calculated by the PCA (Principal Component Analysis, PCA) method. Then, the VFPR model was used to assess the water quality of Biliuhe reservoir from 2013 to 2016. The assessment results are shown in Figure 9.

According to the assessment results, with no thought for sediments release potential, the water quality of Biliuhe reservoir was always between level 2 and level 3 of the National Surface Water Quality Standards of China (GB3838-2002) from 2013 to 2016. When sediments release potential was considered, the water quality showed a decreased tendency with the average value decreasing from 2.5 to 2.9. Among which all the samples in winter and spring were still within level 2 and level 3, but much closer to level 3. Meanwhile, about 42% of the samples in summer and 33% in autumn exceeded level 3.

As can be seen in Figure 9, the water quality of the reservoir was better in winter and spring, but degraded in summer and improved in autumn, which was consistent with previous research [26]. The rainfall of Biliuhe reservoir basin was mainly concentrated in summer and early autumn, so the external pollutants entering the reservoir with rainfall runoff in these two seasons also increased. The decline of water quality in summer and autumn regardless of sediments release potential also proved this hypothesis. The pollutants entering the reservoir were partially deposited at the bottom of the reservoir, increasing the content of pollutants in sediments. In addition, compared with the indicators in winter and spring, the T_W_ in summer and autumn was higher, the DO was lower, and the pH was slightly increased to alkalinity. These factors all contributed to the release of NH_4_^+^–N and TP in the sediments. As a consequence, the water quality was a little worse in summer and early autumn. In winter, the small rainfall, coupled with the barrier of ice, made it difficult for external pollutants to enter the reservoir. Further, because of the purification effect of the reservoir itself, the water quality was the best throughout the year.

## 4. Conclusions

This paper took Biliuhe reservoir, a seasonal reservoir in Northern China, as an example to study the adsorption and release characteristics of the sediments under different environmental conditions, and explored the seasonal variation of water quality considering the release potential of sediments. The main conclusions are as follows. 

(1) Sediments adsorption and release simulation experiments demonstrated that the average concentrations of NH_4_^+^–N and TP in Biliuhe reservoir were lower than the equilibrium concentrations (EC_0_). This indicated that the sediments of Biliuhe reservoir mainly played the “source” of NH_4_^+^–N and TP at present, and the sediments had a higher risk of sediments release with a change in environmental changes in the future.

(2) The release amount of NH_4_^+^–N and TP in the sediments showed a seasonal variation affected by the specific seasonal variation of environmental factors for reservoirs in Northern China. High temperature, low DO, and high pH in summer greatly promoted the release of sediments, which generated the higher release amount in summer than in other seasons. When the release potential of sediments was considered, the average concentration of NH_4_^+^–N increased from 0.28 mg/L to 6.4 mg/L, TP increased from 0.024 mg/L to 0.21 mg/L, and the contents both exceeded level 5 of water quality standards.

(3) The water quality assessment results were degraded when the release potential of sediments was considered, with the average value of 2.5 to 2.9. The degradation was the largest in winter, while it was the smallest in summer. In addition, about 42% and 33% of the samples in summer and autumn exceeded level 3 of water quality standards when considering the release of sediments, indicating that the water in summer and autumn would face greater water quality risks. Therefore, assessment of water quality considering the sediments adsorption and release characteristics is much closer to the reality, especially in summer and winter, which can provide practical guidance for the security of water supply for the reservoir.

## Figures and Tables

**Table 1:** Parameters of the fitting equations about NH_4_^+^–N adsorption and release characteristics in sediments of Biliuhe reservoir. EC, equilibrium concentration.

Indicator	EC_0_ (mg/L)	Langmuir				Freundlich			
		Equation	R^2^	*Q_max_* (mg/kg)	*K_L_* (L/mg)	Equation	R^2^	1/*n*	*K* (L/mg)
NH_4_^+^–N	8.29	1/*q* = 0.0766/*C*+0.0011	0.9735	909.1	0.014	Ln(*q*) = 0.7408ln(*C*)+3.0403	0.9846	0.7408	20.9115

**Table 2:** Parameters of the fitting equations about total phosphorus (TP) adsorption and release characteristics in sediments of Biliuhe reservoir.

Indicator	EC_0_ (mg/L)	Langmuir				Freundlich			
		Equation	R^2^	*Q_max_* (mg/kg)	*K_L_* (L/mg)	Equation	R^2^	1/*n*	*K* (L/mg)
TP	0.025	1/*q* = 0.0077/*C*+0.0032	0.9899	312.5	0.415584	Ln(*q*) = 0.4489ln(*C*)+4.3439	0.9117	0.4489	77.0073
